# Diagnostic challenge in mixed phenotype acute leukemia with T/megakaryocyte or T/myeloid lineages accompanied by t(3;3)

**DOI:** 10.1186/s13000-022-01257-w

**Published:** 2022-10-05

**Authors:** Yannan Jia, Dong Lin, Zhe Wang, Chengwen Li, Huijun Wang, Jianxiang Wang, Yingchang Mi

**Affiliations:** State Key Laboratory of Experimental Hematology, National Clinical Research Center for Blood Diseases, Haihe Laboratory of Cell Ecosystem, Institute of Hematology and Blood Diseases Hospital, Chinese Academy of Medical Sciences and Peking Union Medical College, 300020 Tianjin, China

**Keywords:** Mixed phenotype acute leukemia, Acute megakaryocytic leukemia, EVI-1 rearrangement

## Abstract

**Background:**

The diagnosis of mixed phenotype acute leukemia (MPAL) with T/megakaryocyte or T/myeloid lineages accompanied by t(3;3) is always a challenge. Therefore, multiple experimental methods are usually required to avoid misdiagnosis. In this report, we presented a rare case of MPAL with T/myeloid lineages accompanied by t(3;3) and discussed the experience of differential diagnosis and our appreciation of the MPAL with T/megakaryocyte and T/myeloid lineages accompanied by t(3;3).

**Case presentation:**

A 31-year-old woman was admitted to our hospital due to recurrent fever for 20 days. Two distinct blast populations were detected by flow cytometry analysis: one population fulfills the immunophenotypic criteria for T-lymphoblastic leukemia, while the other population is highly suggestive of megakaryoblasts. These immunophenotypic features support the diagnosis of MPAL (T/megakaryocyte), which is rarely reported​. Interestingly, a complex karyotype was detected afterward by cytogenetics with t(3;3)(q21;q26.2), indicating a diagnosis of AML with t(3;3), a subset of which is also characterized by megakaryocytic markers such as CD41 and CD61. It seems that the second blast population detected by flow cytometry could not be classified into either diagnosis based on the morphology, immunophenotyping, and even cytogenetic findings, posing a real diagnostic problem because of the lack of clear-cut cytogenetic morphological defined criteria to distinguish between acute megakaryocytic leukemia and AML with t(3;3). Combining all of the examination data, this case was ultimately diagnosed as MPAL (T + My)-NOS with t(3;3) through differential diagnosis. Before the cytogenetic results were available, the patient received an acute lymphoblastic leukemia (ALL) regimen for MPAL treatment, but the effect was unsatisfactory. After the diagnosis was clear, she received an AML-like regimen with azacitidine for 7 days and venetoclax for 14 days, and achieved complete morphological remission.

**Conclusion:**

MPAL with either T/megakaryocyte or T/myeloid lineages accompanied by t(3;3) is rare, and it is difficult to make a clear diagnosis. Thus, comprehensive examinations, including bone marrow cell morphology, flow cytometry analysis, cytogenetics, and molecular analysis are recommended to avoid misdiagnosis. AML-like regimen including azacitidine and venetoclax may be effective for treating MPAL (T + My)-NOS with t(3;3).

Ethical lot number: KT2020004-EC-2.

## Background

Mixed phenotype acute leukemia (MPAL) is rare, accounting for 2–5% of all acute leukemia cases [1]. It has blasts that express antigens of more than one lineage and make it impossible to assign leukemia to any one lineage with certainty. MPAL can contain distinct blast populations, with antigen expression most often involving myeloid antigens coexisting with either T-cell or B-cell antigens or more rarely involving both B-cell and T-cell lineages. The diagnosis of MPAL with T/megakaryocyte lineage is exceptionally rare, and both the European Group for the Immunological Classification of Leukemia (EGIL) scoring system and the World Health Organization (WHO) 2016 criteria do not include megakaryoblastic markers in their scoring system. Cases of acute myeloid leukemia (AML) with t(3;3)/inv[3] exhibit similar bone marrow morphology, immunophenotyping, and even cytogenetic phenotype to those of MPAL with T/megakaryocyte lineages, making it difficult to distinguish between them. In this report, we presented a rare case of MPAL with T/myeloid lineages accompanied by t(3;3) and discussed the experience of differential diagnosis and our appreciation of the MPAL with T/megakaryocyte and T/myeloid lineages accompanied by t(3;3).

## Case presentation

**Fig. 1:**
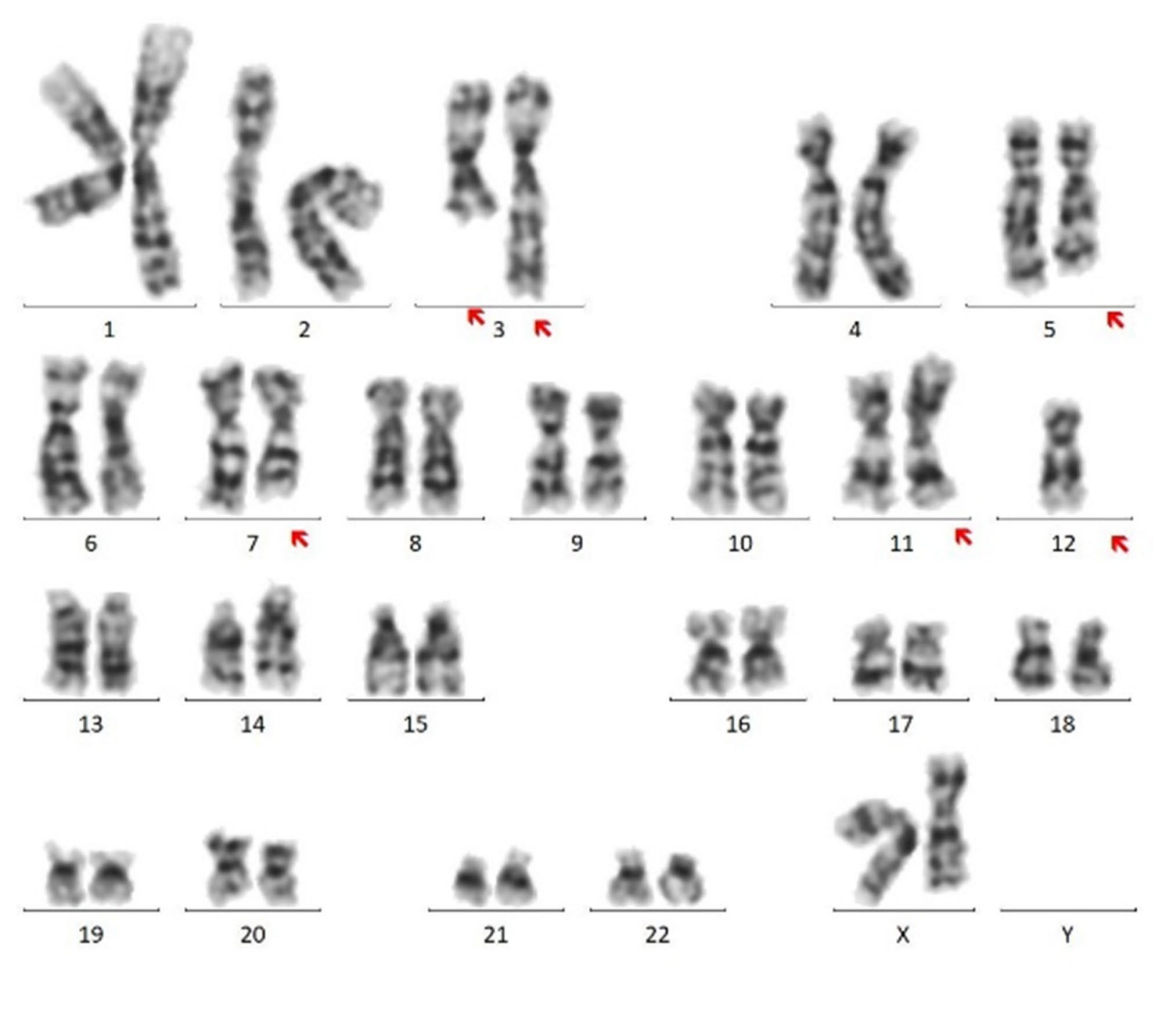
A smear of peripheral blood (PB) shows immature blast cells by Wright Giemsa stain (Panel A: x 100, Panel B: x1000)

**Fig. 2:**
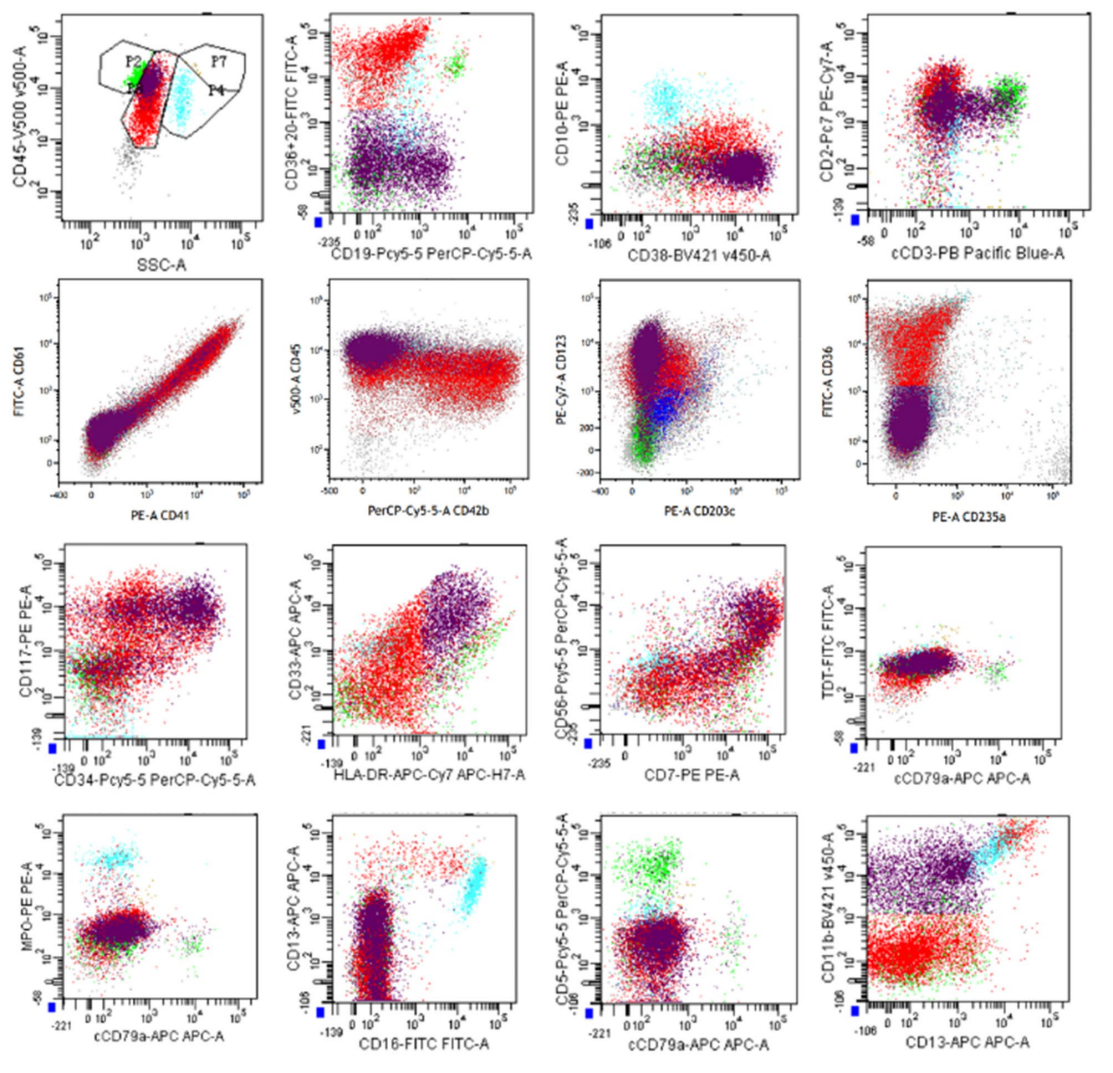
Flow cytometry analysis of peripheral blood. Through a wide monoclonal antibody panel by 8-color flow cytometry analysis, two distinct blast populations were detected (P3). One population occupied 40.3% of all nucleated cells (red), with strong expression of CD38, CD36, CD2, partial expression of CD41, CD61, CD42b, CD34, CD117, CD13, CD123, CD56, CD7, dim expression of HLA-DR, CD33 and absent expression of CD19, CD10, cCD79a, cCD3, CD5, TdT, MPO, CD203c, CD235a. The other population constituted 40.50% of all nucleated cells (purple) and displayed as CD7bri^+^ CD38bri^+^ CD2^+^ CD34^+^ CD117^+^ HLA-DR^+^ CD33^+^ CD11b^+^ CD123^+^ CD56^+^ cCD3^par+^ CD13 ^par+^ CD19 ^par+^ and CD5^−^CD36^−^TdT^−^MPO^−^cCD79a^−^CD19^−^CD10^−^CD203c^−^CD235a^−^CD41^−^CD61^−^CD42b^−^.

**Fig. 3:**
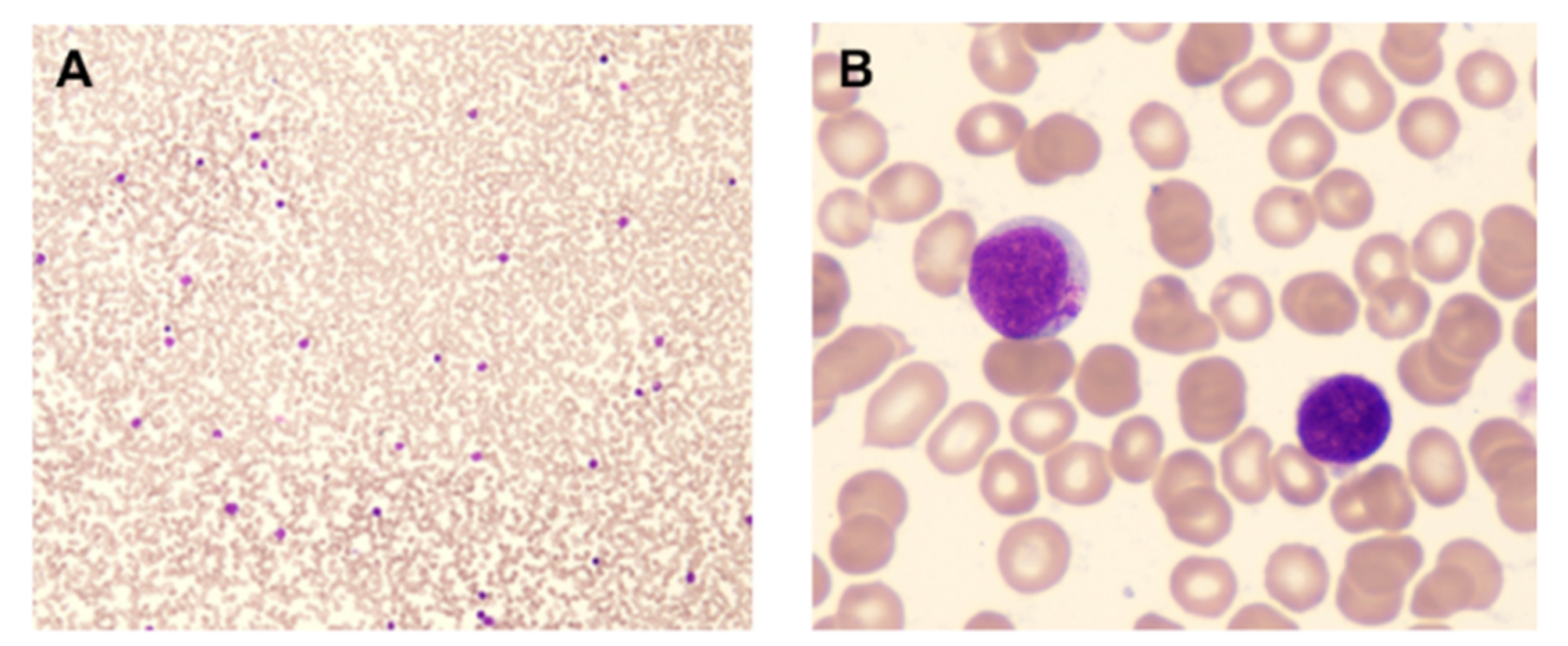
Karyotype analysis of PB sample: 45,XX, t(3;3)(q21;q26.2), add[5](q22), del[7](q31q34), add(11)(p15), -12[20]. Abnormalities in chromosome 3, 5, 7, 11 and 12 (arrows) were detected

**Fig. 4:**
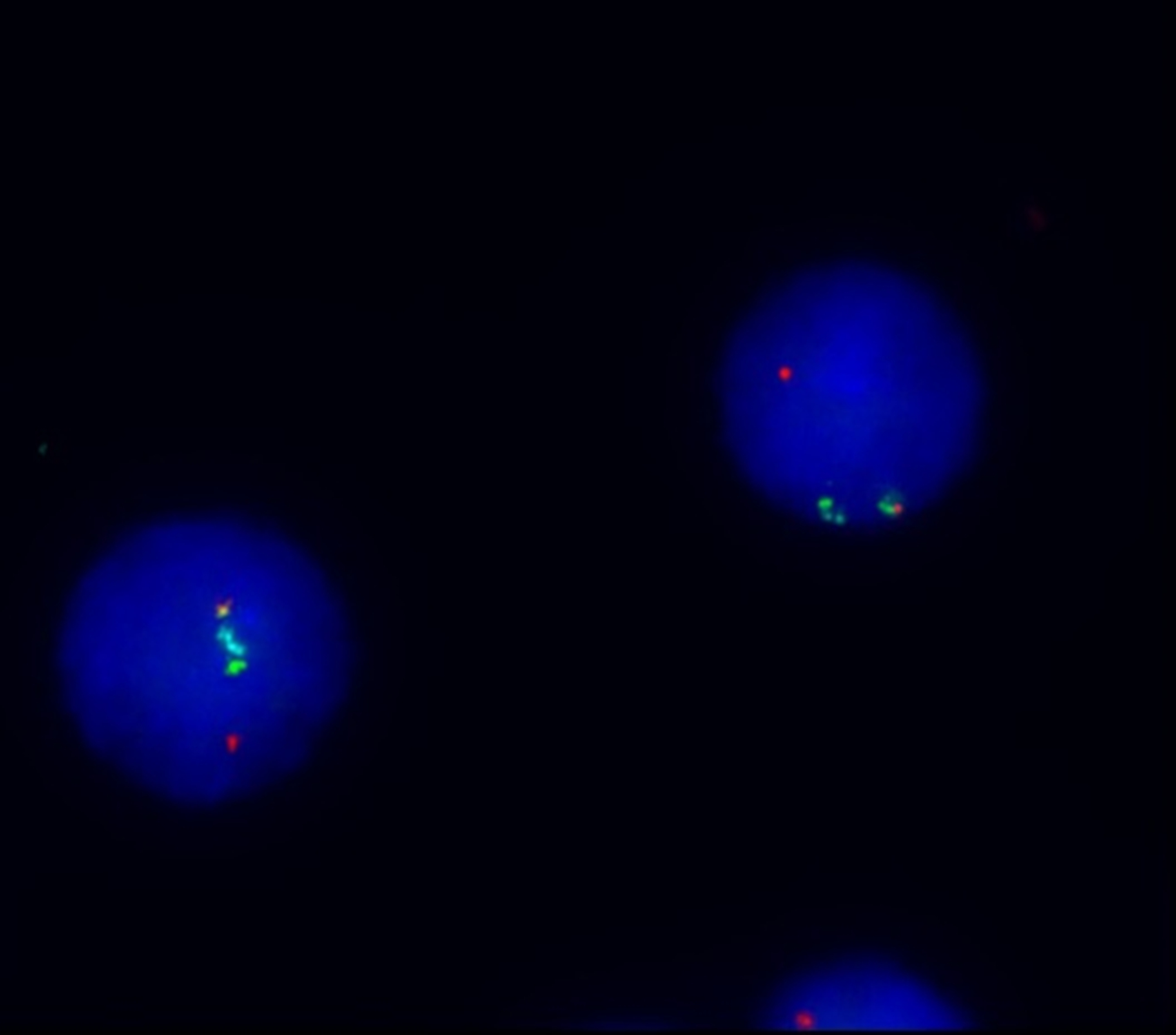
FISH analysis examined by EVI1 probe mix. The red component includes the LRRC34 gene. The green component includes the centromeric part of the EVI1 (MECOM) gene. The blue component covers a 563 kb region centromeric to the EVI1 gene that includes the D3S3364 marker. EVI-1 rearrangement was detected

A 31-year-old woman was admitted to our hospital due to recurrent fever for 20 days. Bone marrow aspirate was checked in our department and showed hypocellularity with 44.5% of blasts. Peripheral blood (59% of blasts, Fig. 1) was used for the following examinations due to bone marrow dry tap. Unexpectedly, two distinct blast populations were detected by flow cytometry analysis (Fig. 2). One population occupied 40.3% of all nucleated cells, with strong expression of CD38, CD36, and CD2, partial expression of CD41, CD61, CD42b, CD34, CD117, CD13, CD123, CD56, and CD7, dim expression of HLA-DR, and CD33, and absent expression of CD19, CD10, cCD79a, cCD3, CD5, TdT, MPO, CD203c, and CD235a, which is highly suggestive of megakaryoblasts. The other population constituted 40.50% of all nucleated cells and was displayed as CD7^bri+^ CD38^bri+^ CD2^+^ CD34^+^ CD117^+^ HLA-DR^+^ CD33^+^ CD11b^+^ CD123^+^ CD56^+^ cCD3^par+^ CD13 ^par+^ CD19 ^par+^ and CD5^−^ CD36^−^ TdT^−^ MPO^−^ cCD79a^−^ CD19^−^ CD10^−^ CD203c^−^ CD235a^−^ CD41^−^ CD61^−^ CD42b^−^, which fulfills the immunophenotypic criteria for T-lymphoblastic leukemia. These immunophenotypic features support the diagnosis of MPAL (T/megakaryocyte), which is rarely reported​. Interestingly, a complex karyotype (Fig. 3) was detected by cytogenetics with t(3;3)(q21;q26.2), indicating a diagnosis of AML with t(3;3), a subset of which is also characterized with megakaryocytic markers such as CD41 and CD61. Besides, the cytochemical staining with CD41 antibody showed 17% CD41^+^ cells, including 6% megakaryoblasts and 11% micromegakaryocytes. Molecular analysis was negative for 56 commonly expressed fusion genes and FLT-3 and WT-1 alterations. Fluorescence in situ hybridization (FISH) examination identified a strong positivity (93%) of EVI-1 rearrangement (Fig. 4) whose overexpression was further confirmed by polymerase chain reaction test. Furthermore, TCR-γ rearrangement was also positive and IGH rearrangement was negative. Next-generation sequencing screening revealed CCND3 (VAF:19.90%), KRAS (VAF:66.00%), and APC (VAF:81.70%) mutations and no genetic abnormalities involved in BCL11B was identified. Before the cytogenetic results were available, she received an ALL regimen for MPAL treatment with vincristine, daunorubicin, cyclophosphamide, and prednisone. However, the effect of this VDCP scheme treatment was unsatisfactory, and the percentages of two original blast populations examined by flow cytometry remained unchanged on day 15. When the diagnosis was clear, she received an AML-like regimen with azacitidine for 7 days and venetoclax for 14 days. After treatment regimen adjustment, she achieved complete remission with no blast cells identified in the bone marrow.

## Discussion

MPAL can contain distinct blast populations of different lineages or one population with multiple antigens of different lineages. This case fits into the former category as two atypical blast populations were detected by flow cytometry. As shown in Fig. 2, the cCD3^par+^CD7^bri+^CD2^+^ expression profile and TCR-γ rearrangement in one of the populations are in accordance with the T-lymphoblastic leukemia phenotype. Although the cCD3 was partially expressed, the brightest cCD3-positive blasts reached the intensity of the normal residual T cells presented in this sample, fulfilling the immunophenotypic criteria for T-lymphoblastic leukemia according to the WHO 2016 criteria. While the other population posed a real problem of diagnosis for us because of the lack of clear-cut cytogenetic morphological defined criteria to distinguish between AML (AML-M7) and AML with t(3;3).

AMKL or AML-M7 as defined by FAB classification is a rare subtype of AML [2]. A limited number of patients had been diagnosed with AMKL due to its low incidence. For the majority of AMKL cases, morphological aspect of blast cells was very megakaryocytic specific, allowing these cells to be classified as megakaryoblasts. However, it should be noted that, in some AMKL cases, all the blast cells could be undifferentiated or with abnormal megakaryocytic maturation and essential micromegakaryocytes. Although no typical megakaryoblasts were identified in this case, the diagnosis of AMKL should not be excluded. Therefore, the immunophenotyping analysis for a megakaryoblastic assignment was required due to the wide morphology heterogeneity with AMKL. The immunological identification of megakaryoblasts mainly depends on the detection of specific lineage markers, such as CD41, CD42, and CD61, according to immunologic recommendations [3]. In this case, the other blast population uniformly expressed such markers, highlighting a megakaryoblast subtype. Although in some cases, the expression of CD41 or CD61 can be misinterpreted as positive staining on flow cytometry due to possible adherence of platelets to blast cells, the consistent positive CD41 expression on cytochemical staining in this sample can exclude this possibility.

MPAL-NOS T/megakaryocytic type is exceptionally rare. To date, there have been very few reports of B or T/megakaryocyte. Gupta et al. reported a rare case diagnosed as MPAL-NOS (T/megakaryocyte) with a complex karyotype [4]. In their case, the positivity of CD61 and cCD3 was examined in two separate populations by immunohistochemical staining instead of flow cytometry. With two cycles of AML chemotherapy and subsequent bone marrow hematopoietic stem cell transplantation, the patient survived and returned to normal life.

Of note, megakaryocytes are not specific for AML-M7. Some other cases are also accompanied by increased or abnormal megakaryocytes, including patients with AML with t(3;3)(q21.3;q26.2) or inv[3](q21.3q26.2). As the cytogenetic results came out, the previously suspected diagnosis was challenged. The other blast population could also be considered AML with t(3;3)(q21.3;q26.2), but not AML-M7. Besides, the high positivity of EVI-1 rearrangement by FISH examination showed that this blast population possibly derive from one major clone.

Cases of AML with t(3;3)/inv[3] are rare and account for 1–2.5% of all AML and 1% of MDS, resulting in deregulated MECOM (also called EVI1) and GATA2 expression [5]. Trilineage dysplasia is common, and increased or atypical bone marrow megakaryocytes are the most frequent and characteristic feature in these cases, which are sometimes even morphologically recognized as AML-M7 [6–8]. Notably, a subset of such cases can also express megakaryocytic markers such as CD41 and CD61. In this case, the increased small micromegakaryocytes by cytochemical staining, as well as the flow cytometry and cytogenetics data, are all consistent with the diagnosis of MPAL with t(3;3). A series of 61 patients with AML with inv[3]/t(3;3) reported by Sitges et al. showed that dysmegakaryopoiesis was more frequent than dysgranulopoiesis or dyserythropoiesis. In their study, one patient with inv[3], but not t(3;3), was diagnosed with acute leukemia of ambiguous lineage and received acute lymphoblastic leukemia-based induction therapy [5].

It seems that the second blast population detected by flow cytometry could not be classified into either diagnosis based on the morphology and immunophenotyping data. So, could the cytogenetic finding of t(3;3) do the job? The answer may be no. Although AMKL is not characterized by any specific chromosome abnormality, it may represent at least three separate disease entities. The first is pediatric patients with Down syndrome AML. The second is patients with t(1;22)(p13;q13) or other cytogenetic abnormalities involving 22q13. The third is the remaining patients with AMKL who can carry cytogenetic abnormalities in chromosome 3 [Monosomy 3, Inv[3](q21q26) and t(3;21)] [9]. However, based on the WHO criteria for AMKL, the diagnosis for this category should exclude cases with recurrent genetic abnormalities, including AML associated with t(3;3)(q21.3;q26.2). Therefore, the diagnosis of MPAL with T/megakaryocyte is no longer considered.

14q32/BCL11B rearrangement is identified in ~ 20–30% of T/myeloid MPAL, and is increasingly recognized as the cytogenetic hallmark of this entity. The new WHO classification has classified such cases as acute leukaemia of ambiguous lineage with BCL11B rearrangement for its importance [10]. Given that BCL11B is involved with 14q32 translocations, we re-checked the karyotyping data, but no translocation involved in 14q32 was found. We further checked the RNA sequencing data, consistent with cytogenetic results, no fusion transcripts involving BCL11B were detected. Combining all of the examination data, this case was ultimately diagnosed as MPAL (T + My)-NOS with t(3;3).

## Conclusion

MPAL with either T/megakaryocyte or T/myeloid lineages accompanied by t(3;3) is rare, and it is difficult to make a clear diagnosis. Thus, comprehensive examinations, including bone marrow cell morphology, flow cytometry analysis, cytogenetics, and molecular analysis, are recommended to avoid misdiagnosis. AML-like regimen including azacitidine and venetoclax may be effective for treating MPAL (T + My)-NOS with t(3;3).

## Data Availability

The data used during the current study is available from the corresponding author on reasonable request.

## Abbreviations

ALL: Acute lymphoblastic leukemia.
AML: Acute megakaryocytic leukemia.
EGIL: European Group for the Immunological Classification of Leukemia.
FISH: Fluorescence in situ hybridization.
MPAL: Mixed phenotype acute leukemia.
WHO: World Health Organization.
